# Chemotherapy Combined With Immunotherapy as a First-Line Treatment Brings Benefits to Patients With Lung Squamous Cell Carcinoma but Different Risks of Adverse Reactions: A Systematic Review and Meta-Analysis

**DOI:** 10.3389/fphar.2022.940567

**Published:** 2022-07-01

**Authors:** Qian Chen, Zhen Zhang, Xiaoli Li, Lingbiao Bu

**Affiliations:** ^1^ Department of Pharmacy, Beijing Gaobo Boren Hospital, Beijing, China; ^2^ Department of Anesthesiology, Zoucheng People’s Hospital, Jining, China

**Keywords:** immunotherapy, chemotherapy, squamous NSCLC, overall survival, progression-free survival, security

## Abstract

**Objective** To explore the efficacy and safety of chemotherapy combined with immunotherapy as the first-line treatment of advanced or metastatic squamous NSCLC.

**Methods** Two researchers independently searched PubMed, the Cochrane Library, EMBASE, CNKI, Wanfang Data, and other databases by using a computer, collected the clinical trials or randomized controlled trials published by April 2022 about immunotherapy combined with chemotherapy as the first-line treatment of advanced or metastatic squamous NSCLC, screened the literature, and extracted the data according to the nanodischarge criteria. We used Revman5.4 for statistical analysis of the included studies, and publication bias was analyzed with Egger’s test in Stata12.

**Results** A total of seven clinical trials were included, including 1,510 cases in the chemotherapy combined with the immunotherapy group and 1,519 cases in the chemotherapy group. In terms of effectiveness, compared with the chemotherapy group, chemotherapy combined with immunotherapy for advanced or metastatic squamous NSCLC had longer overall survival (HR = 1.59, 95% CI: 1.46–1.72, *p* < 0.00001) and progression-free survival (HR = 1.84, 95% CI: 1.66–2.03, *p* < 0.00001). In terms of safety, the chemotherapy combined with immunotherapy group has a higher risk of adverse reactions at any level and above three levels of hematotoxicity, gastrointestinal abnormalities, and liver dysfunction than the chemotherapy group. Egger’s test has minor publication bias.

**Conclusion** Chemotherapy combined with immunotherapy is effective as the first-line treatment for advanced or metastatic squamous NSCLC, but the risk of adverse reactions is relatively high. If there are adverse reactions in clinical application, it should be treated in time.

**Clinical Trial Registration:** https://Systematicreview.gov/, identifier [registration number]

## Introduction

At present, the mortality rate of lung cancer is still the highest among malignant cancers in China, and the 5-year survival rate is about 20.5% (Chen et al., 2016). Lung cancer is divided into non–small cell lung cancer and small cell lung cancer. Non–small cell lung cancer accounts for more than 85% of lung cancer cases. It is the most common lung cancer. It can be divided into adenocarcinoma and squamous cell carcinoma. Squamous non–small cell lung cancer (sq-NSCLC) accounts for about 30% of NSCLC cases. It is a very serious type of lung cancer with difficult treatment and poor prognosis. In the past 20 years, many effective treatment options have been limited to non-squamous NSCLC. There are serious unmet clinical needs for sq-NSCLC, and patients with advanced sq-NSCLC urgently need more new treatment options. Because the sensitivity of sq-NSCLC to chemotherapeutic drugs is significantly lower than that of adenocarcinoma non–small cell lung cancer, some NSCLC treatment schemes (including new targeted oncogene drugs, chemotherapeutic drugs, and anti-angiogenesis therapy) have limitations in the effectiveness or safety of sq-NSCLC (American Association for Cancer Research, 2016). Therefore, at present, the first-line treatment used by the vast majority of sq-NSCLC patients is still the combined chemotherapy containing platinum drugs. However, with continuous maturity of gene detection technology, gene-targeted therapy and immunotherapy have been applied in clinics (Rossi and Di Maio, 2016).

Tumor immunotherapy is to control and kill tumor cells by mobilizing the function of the body’s immune system and enhancing antitumor immunity. It is the most effective antitumor treatment after surgery, radiotherapy, chemotherapy, and targeted therapy (Seelige et al., 2018). In recent years, immune checkpoint inhibitors such as PD1/PD-L1 have become a research hotspot of immunotherapy, and they have also achieved a breakthrough in clinical treatment. At present, drugs such as sintilimab, tisliezumab, pembrolizumab, and camrelizumab combined with conventional chemotherapy in the treatment of sq-NSCLC have achieved excellent performance, but there is a lack of systematic evaluation of its clinical effect and safety. This study systematically reviewed several drugs commonly used in sq-NSCLC immunotherapy, evaluated the efficacy and safety of chemotherapy combined with immunotherapy as the first-line treatment of advanced or metastatic squamous NSCLC, and provided the basis for clinical treatment.

## Materials and Methods

We systematically searched for global clinical trials or RCTs of chemotherapy combined with immunotherapy as the first-line treatment of advanced or metastatic squamous NSCLC and systematically evaluated its efficacy and safety.

Inclusion criteria: 1) included population: patients with advanced or metastatic sq-NSCLC who received chemotherapy combined with immunotherapy; 2) literature: retrospective study and prospective study; 3) intervention measures: the treatment group was given chemotherapy combined with immunotherapy (platinum combined with paclitaxel, platinum combined with protein-bound paclitaxel, platinum combined with gemcitabine, and other conventional first-line chemotherapy drugs), and the control group was given placebo combined with chemotherapy or conventional chemotherapy; 4) OUTCOME MEASURES: the main outcome measures included progression-free survival (PFS) and overall survival (OS)–related hazard ratio (HRS) and 95% confidence interval (95% CI). Secondary outcome measures included adverse reactions at any level (hematotoxicity, gastrointestinal abnormalities, and liver dysfunction) and adverse reactions above level 3 (hematotoxicity, gastrointestinal abnormalities, and liver dysfunction).

Exclusion criteria: 1) no control group was established; 2) repeatability study; 3) non-Chinese and English literature; 4) HR literature for PFS and/or OS were not provided.

### Literature Screening and Data Extraction

We used a variety of search tools to conduct a comprehensive search of the literature. 1) Retrieval of the computer literature database: ① key words include immunotherapy, sintilimab, tisliezumab, pembrolizumab, camrelizumab, sq-NSCLC, programmed cell death protein 1, programmed cell death protein ligand 1, clinical trials, RCTs, etc.,② Search PubMed, Cochrane Library, EMBASE, and CNKI in the form of keyword joint free words. Wanfang database, etc., the retrieval time limit is from the establishment of the database to April 2022. 2) manual retrieval of ASCO conference-related literature as a supplement to computer retrieval.

The extracted data mainly include the following: author’s name, publication year, patient sample size, treatment methods, HRs, and 95% CI of PFS and OS.

### Bias Risk Assessment

The Newcastle Ottawa scale (NOS) was used to evaluate the literature quality (Lazarus et al., 2019), and the quality of the included studies was evaluated according to the following eight criteria: 1) the representativeness of the exposure cohort; 2) selection of non-exposed cohorts; 3) determination of exposure methods; 4) there were no outcome events before the start of the study; 5) comparability between exposed and non-exposed cohorts; 6) evaluation of outcome events; 7) whether the follow-up time is long enough; 8) whether the follow-up is complete. Literature with a score of 7–9 is considered “high,” 4–6 is “average,” and 3 or lower is “low”. The quality evaluation shall be carried out independently and cross-checked by two researchers. In case of differences, the third researcher should help solve them.

### Statistical Analysis

We used Revman 5.4 software provided by the Cochrane Collaboration Network for meta-analysis. All HRs included in the study were brought together to provide the overall effect size. The Cochrane χ^2^ test was used to analyze the heterogeneity among studies, and I^2^ was used to evaluate the heterogeneity. When *p* > 0.1 and I^2^ < 50%, it indicates that there is no statistical heterogeneity in each RCTs, and the fixed-effect model is used; on the contrary, on the premise of excluding clinical heterogeneity, the random-effect model is used. The publication bias was analyzed by an inverted funnel diagram, the sensitivity of each included literature was analyzed, and the test bias of the included literature was discussed.

## Results

### Literature Search and Screening

**FIGURE 1:**
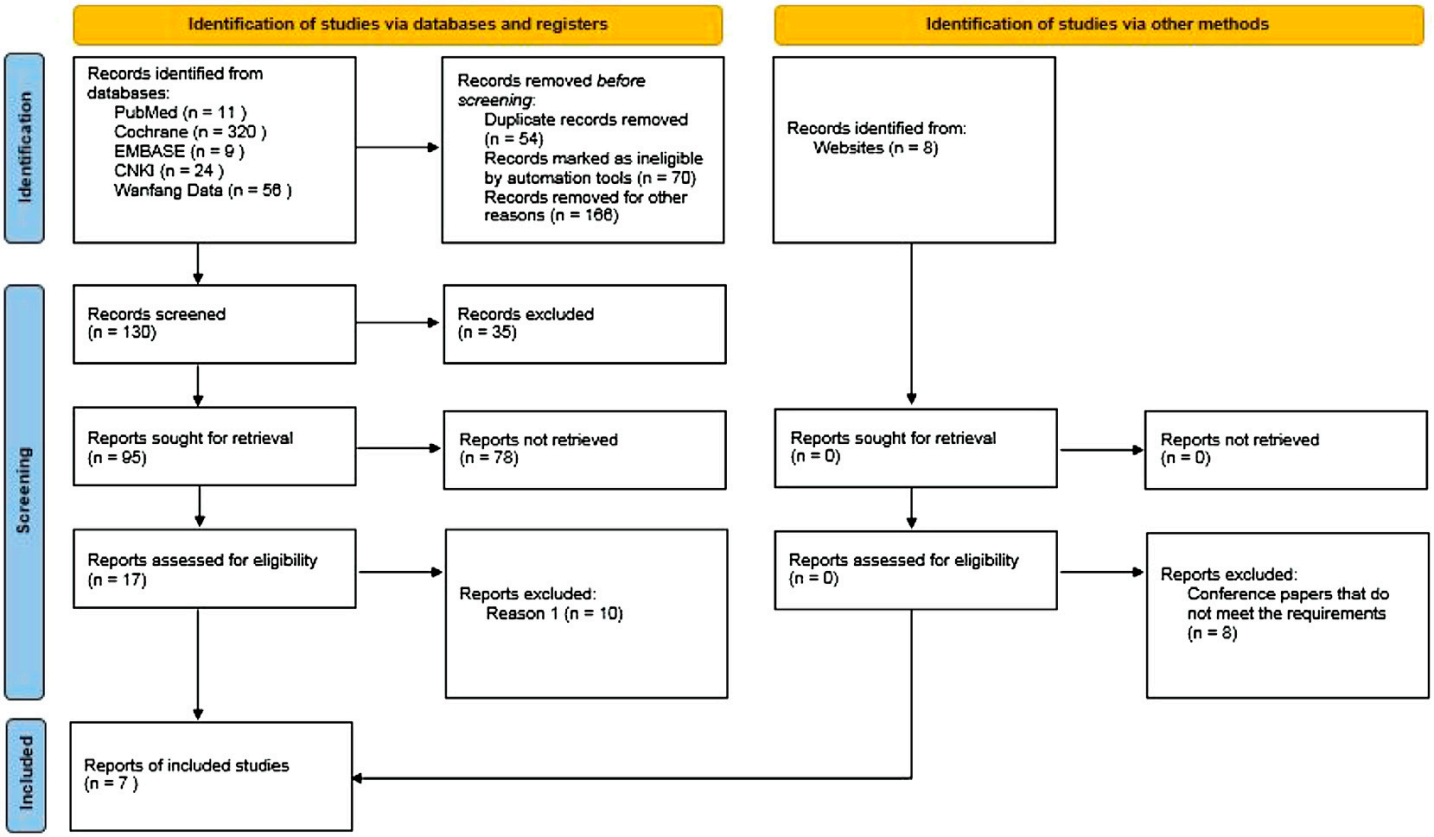
PRISMA flow chart of article selection.

Through database retrieval, there were 11 literatures in PubMed, 320 literature in the Cochrane Library, nine literatures in EMBASE, 24 literatures in CNKI, and 56 literatures in Wanfang database, and a total of 420 relevant literatures and eight conference papers and abstracts were obtained. After excluding duplication, case reports, reviews and irrelevant contents, 130 literatures were screened in strict accordance with the abovementioned screening process, and finally seven (Halmos et al., 2018; Paz-Ares et al., 2018; Cheng et al., 2019; Paz-Ares et al., 2020; Wang et al., 2021; Zhou et al., 2021; Ren et al., 2022) studies were included, which met the quantitative analysis, as shown in Figure 1. A total of 3,029 cancer patients who met the requirements were included in the seven literatures, of which 1,510 patients received chemotherapy combined with immunotherapy and 1,519 patients received routine chemotherapy. All seven literatures are high-quality literatures, as shown in Table 1.

**TABLE 1:** Basic characteristics of included studies.

First author	Year	Phase	NO. of immune + chemo	NO. of chemo	Protocol of immune + chemo	Protocol of chemo	HR for PFS [95% CI]	*p*-Value for PFS	HR for OS [95% CI]	*p*-Value for OS	Quality
Caicun Zhou (Zhou et al., 2021)	2021	III	179	178	Sintilimab + PG	placebo + PG	0.536 [0.422.0.681]	<0.00001	0.567 [0.353.0.909]	0.01701	7
Jie Wang Wang et al. (2021)	2021	III	120	121	Tislelizumab + PC	PC	0.52 [0.37.0.74]	<0.001	NA	NA	8
	2021	III	119	121	Tislelizumab + nab-PC	nab-PC	0.48 [0.34.0.68]	<0.001	NA	NA	8
L. Paz-Ares Paz-Ares et al. (2018)	2018	III	278	281	Pembrolizumab + PC	placebo + PC	0.56 [0.45.0.70]	<0.001	0.64 [0.49.0.85]	<0.001	8
Luis Paz-Ares Paz-Ares et al. (2020)	2020	III	278	281	Pembrolizumab + PC	placebo + PC	0.57 [0.47.0.69]	<0.001	0.71 [0.58.0.88]	<0.001	7
Shengxiang Ren Ren et al. (2022)	2021	III	193	196	Camrelizumab + PC	placebo + PC	0.37 [0.29.0.47]	<0.0001	0.55 [0.40.0.75]	<0.0001	8
B. Halmos Halmos et al. (2018)	2018	III	169	167	Pembrolizumab + PC	placebo + PC	0.52 [0.40.0.68]	NA	0.67 [0.48.0.93]	NA	8
	2018	III	109	114	Pembrolizumab + nab-PC	placebo + nab-PC	0.65 [0.45.0.94]	NA	0.59 [0.36.0.98]	NA	7
Y. Cheng Cheng et al. (2019)	2019	III	65	60	Pembrolizumab + PC	placebo + PC	0.32 [0.21.0.49]	NA	0.44 [0.24.0.81]	NA	7

PFS, progression-free survival; OS, overall survival; HR, hazard ratio; NA, not available; PG, Platinum + Gemcitabine; PC, paclitaxel + carboplatin; nab, nanoparticlealbumin-bound.

### Meta-Analysis Results of Efficacy

**FIGURE 2:**
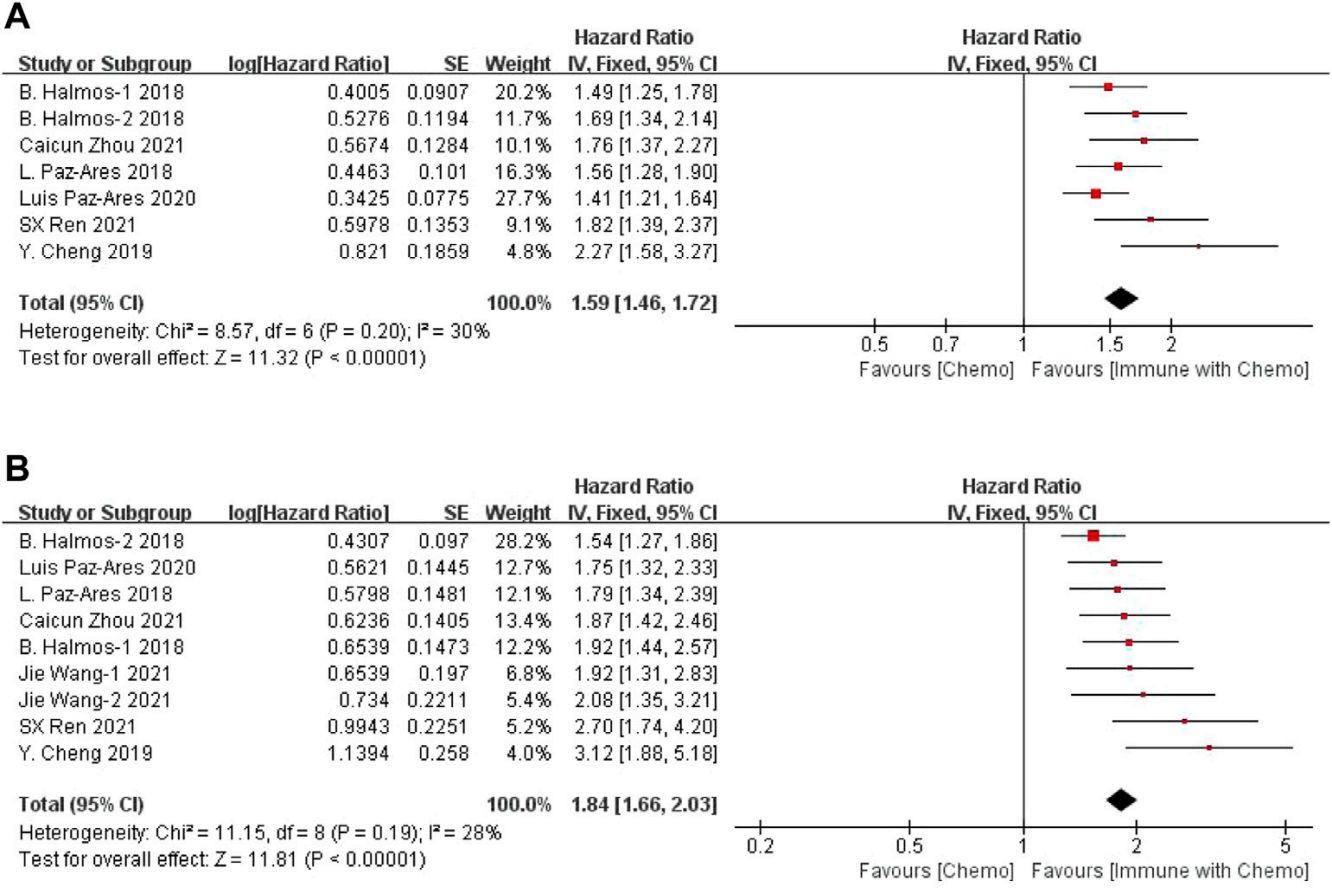
Meta-analysis results of OS **(A)** and PFS **(B)** between the chemo with immune group and chemo group.

The results of the OS analysis can be obtained from seven groups of data in the seven included studies. For heterogeneity analysis, I^2^ = 30%, *p* = 0.20. There is no statistical heterogeneity among the studies. The fixed-effect model is used for analysis. The results showed that HR = 1.59 (95% CI = 1.46–1.72, *p* < 0.00001), suggesting that chemotherapy combined with immunotherapy can significantly prolong the overall survival of patients in the treatment of sq-NSCLC, as shown in Figure 2A. PFS data were obtained from nine groups of data. Heterogeneity analysis showed that I^2^ = 28%, *p* = 0.19. There was no statistical heterogeneity among studies. A fixed-effect model was used for analysis. The results showed that HR = 1.84 (95% CI = 1.66–2.03, *p* < 0.00001), suggesting that immunotherapy combined with chemotherapy can significantly prolong the progression-free survival of patients in the treatment of sq-NSCLC, as shown in Figure 2B.

### Meta-Analysis Results of Adverse Reactions at Any Level

#### Meta-Analysis Results of Hematological Toxin

**FIGURE 3:**
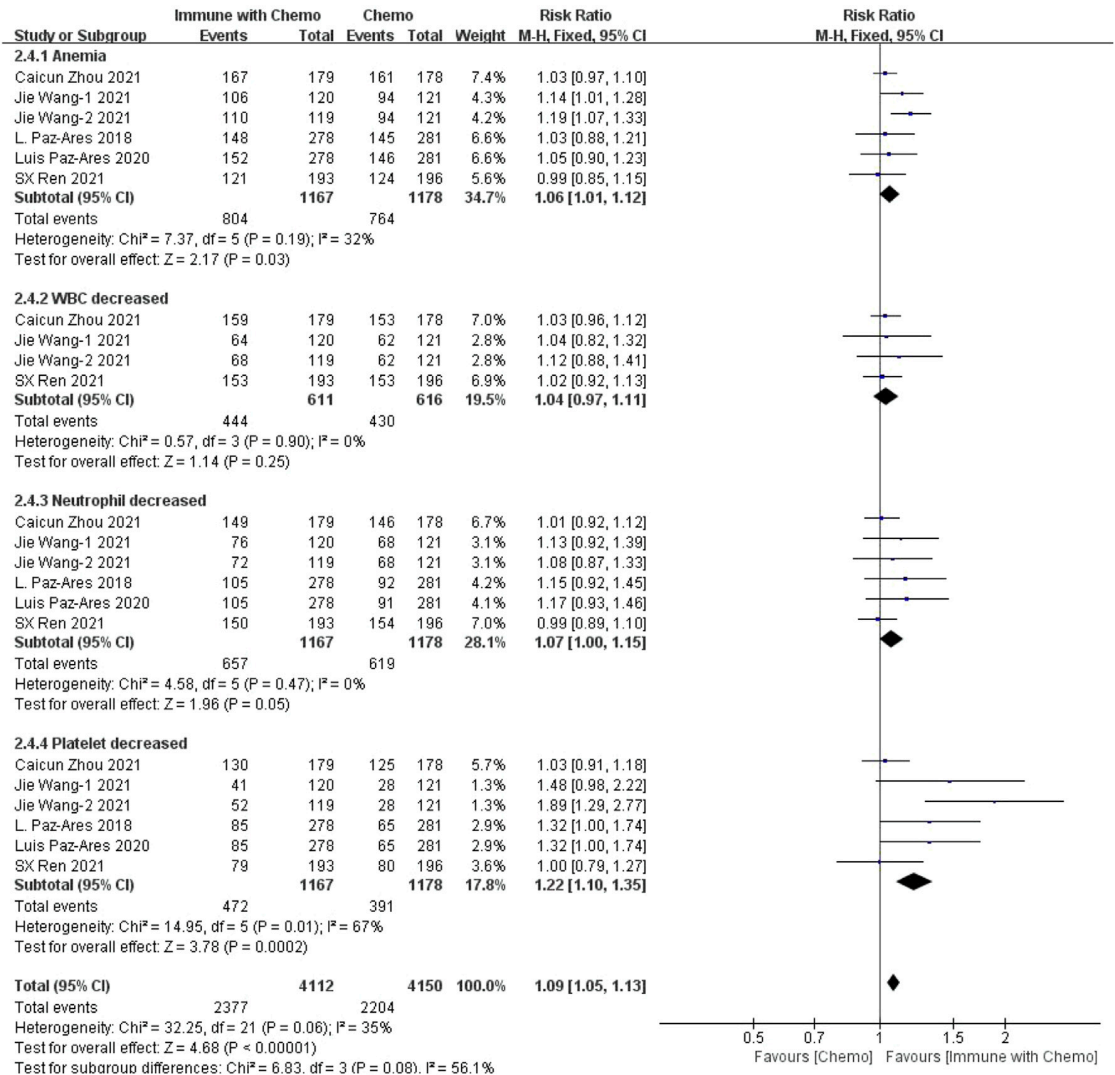
Meta-analysis results of adverse reactions of any grade in hematological toxicity between the chemo with immune group and chemo group.

Six studies can obtain the data of any level of adverse reactions in the blood system of sq-NSCLC patients treated with chemotherapy combined with immunotherapy (including anemia, white blood cell (WBC) discrete, neutrophil discrete, and platelet discrete). The heterogeneity analysis is carried out, with I^2^ = 35%, *p* = 0.06, which is analyzed by the fixed-effect model. The results showed that HR = 1.09 (95% CI = 1.05–1.13, *p* < 0.00001), suggesting that the incidence of adverse reactions at any level of the blood system using chemotherapy combined with immunotherapy is relatively high, and there are significant differences in individual results, as shown in Figure 3. In the subgroup analysis of this study, there was significant heterogeneity in thrombocytopenia indicators (I^2^ = 67%). With factor by factor exclusion, Caicun Zhou (Zhou et al., 2021) and SX Ren (Ren et al., 2022) were found to be the sources of heterogeneity. The different medication regimens of the two experimental groups caused high heterogeneity, and I^2^ was 0% after exclusion.

#### Meta-Analysis Results of Gastrointestinal Toxin

**FIGURE 4:**
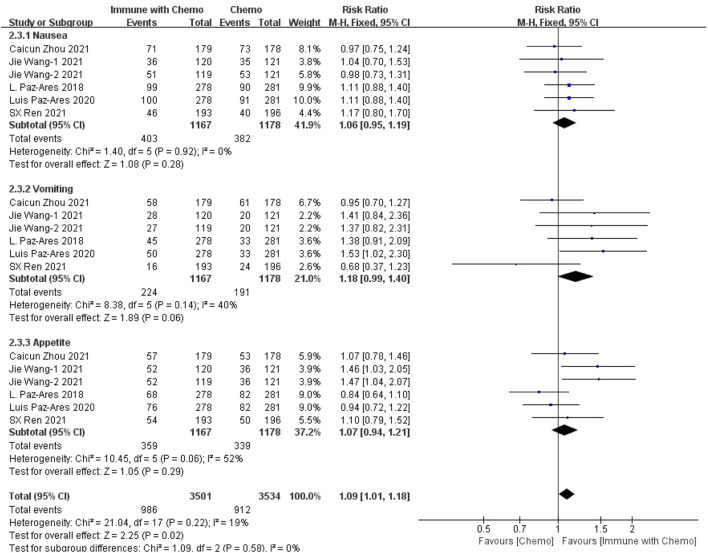
Meta-analysis results of adverse reactions of any grade in gastrointestinal toxicity between the chemo with immune group and chemo group.

Six studies can obtain data on any level of adverse reactions in the gastrointestinal system in patients with sq-NSCLC treated by chemotherapy combined with immunotherapy (including nausea, vomiting, and appetite). The heterogeneity analysis is carried out, with I^2^ = 19%, *p* = 0.22, which is analyzed by the fixed-effect model. The results showed that HR = 1.09 (95% CI = 1.01–1.18, *p* = 0.02), suggesting that the incidence of adverse reactions at any level of the gastrointestinal system caused by chemotherapy combined with immunotherapy is relatively high, but there is no significant difference in the overall results, as shown in Figure 4. In the subgroup analysis of this study, significant heterogeneity exists in the appetite indicator (I^2^ = 52%). With factor by factor exclusion, L. Paz-Ares (Paz-Ares et al., 2018) and Luis Paz-ares (Paz-Ares et al., 2020) are the sources of heterogeneity. Compared with other studies, in both studies, adverse reactions to appetite were lower in combination with the immunochemotherapy regimen than in the control group, and I^2^ heterogeneity was 6% after exclusion.

#### Meta Analysis Results of Hepatotoxicity

**FIGURE 5:**
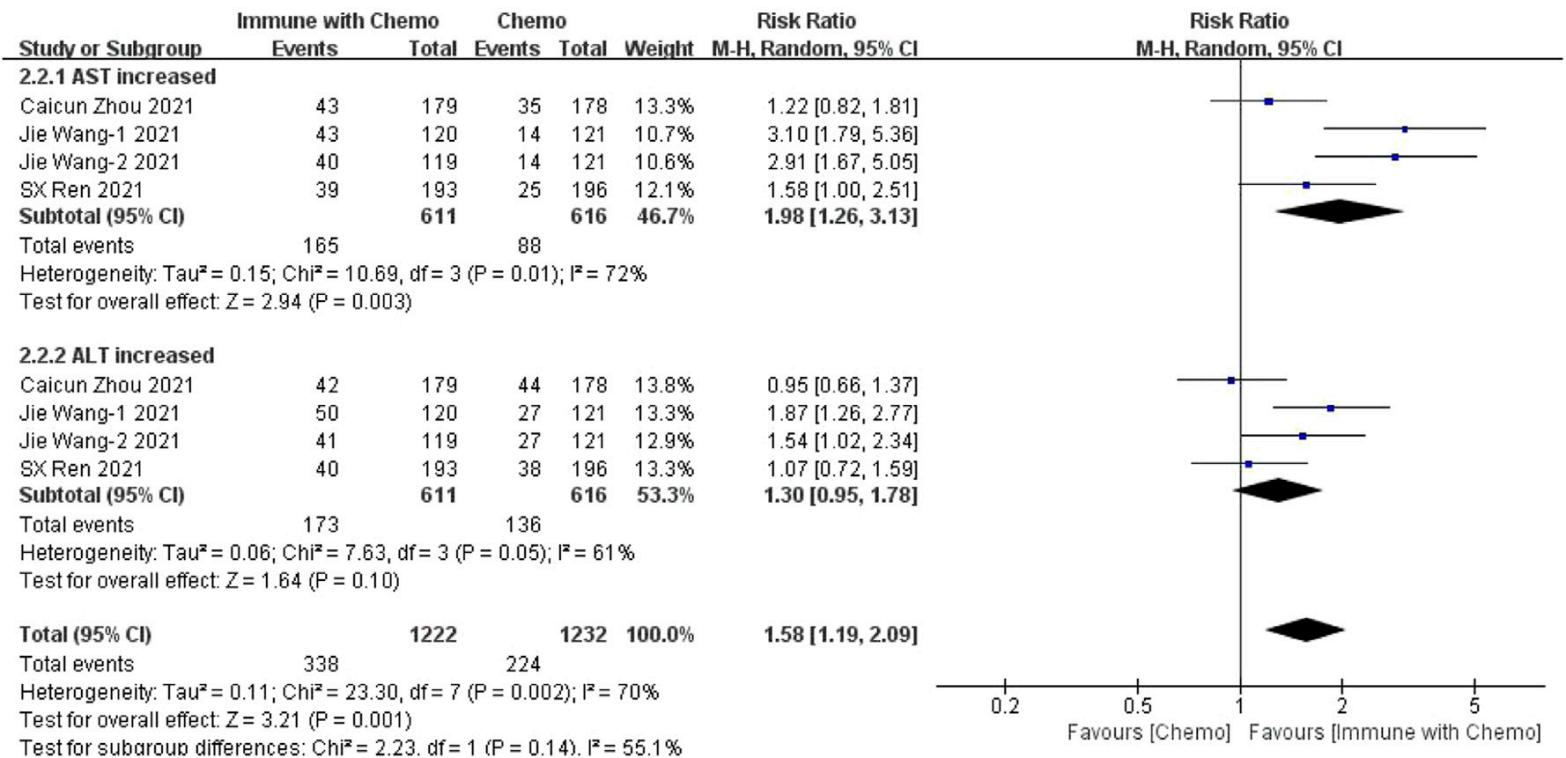
Meta-analysis results of adverse reactions of any grade in hepatotoxicity between the chemo with immune group and chemo group.

Four studies can obtain the data of any level of adverse reactions (including aspartate aminotransferase (AST) and alanine aminotransferase (ALT)) of liver function in patients with sq-NSCLC treated by chemotherapy combined with immunotherapy. The heterogeneity was analyzed, with I^2^ = 70%, *p* = 0.002. The random-effect model is used for analysis. Four studies can obtain the data of any level of adverse reactions (including AST and ALT) of liver function in patients with sqnsclc treated by chemotherapy combined with immunotherapy. The heterogeneity is analyzed, with I^2^ = 70%, *p* = 0.002. The random-effect model is used for analysis. The results showed that HR = 1.58 (95% CI = 1.19–2.09, *p* = 0.001), suggesting that the incidence of adverse reactions at any level of liver function using chemotherapy combined with immunotherapy is relatively high, and there is a significant difference in the results, as shown in Figure 5. In the subgroup analysis of this study, there was significant heterogeneity in AST (I^2^ = 72%) and ALT (I^2^ = 61%) indexes. With factor by factor exclusion, Caicun Zhou (Zhou et al., 2021) and SX Ren (Ren et al., 2022) were found to be the sources of heterogeneity. In the two studies, different drug regimens in the experimental group caused high heterogeneity compared with other groups, and the heterogeneity I^2^ of AST and ALT was 0% after exclusion.

### Meta-Analysis Results of Adverse Reactions Above Grade 3

#### Meta-Analysis Results of Hematological Toxin

**FIGURE 6:**
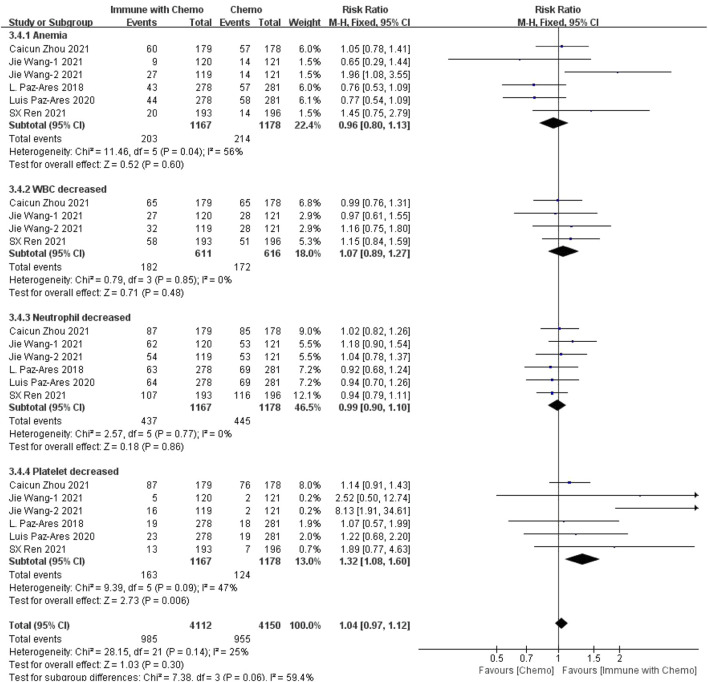
Meta-analysis results of adverse reactions above grade 3 in hematological toxicity between the chemo with immune group and chemo group.

Six studies can obtain the data of hematological grade III or above adverse reactions (including anemia, WBC increase, neutrophil increase, and platelet increase) of patients with sq-NSCLC treated by chemotherapy combined with immunotherapy. The heterogeneity was analyzed with I^2^ = 25%, *p* = 0.14, which was analyzed by the fixed-effect model. The results showed that HR = 1.04 (95% CI = 0.97–1.12, *p* = 0.30), suggesting that the incidence of adverse reactions above grade III in the blood system using chemotherapy combined with immunotherapy is relatively high, but there is no significant difference in the overall results, as shown in Figure 6. In the subgroup analysis of this study, there is significant heterogeneity in the Cia index (I^2^ = 56%). Factor by factor elimination, Caicun Zhou (Zhou et al., 2021), SX Ren (Ren et al., 2022) and Jie Wang-2 (Wang et al., 2021) are the sources of heterogeneity. The immunization combined with chemotherapy in the three studies had a high indemnity compared with the control group. The heterogeneity I^2^ was 0% after the exclusion.

#### Meta-Analysis Results of Gastrointestinal Toxin

**FIGURE 7:**
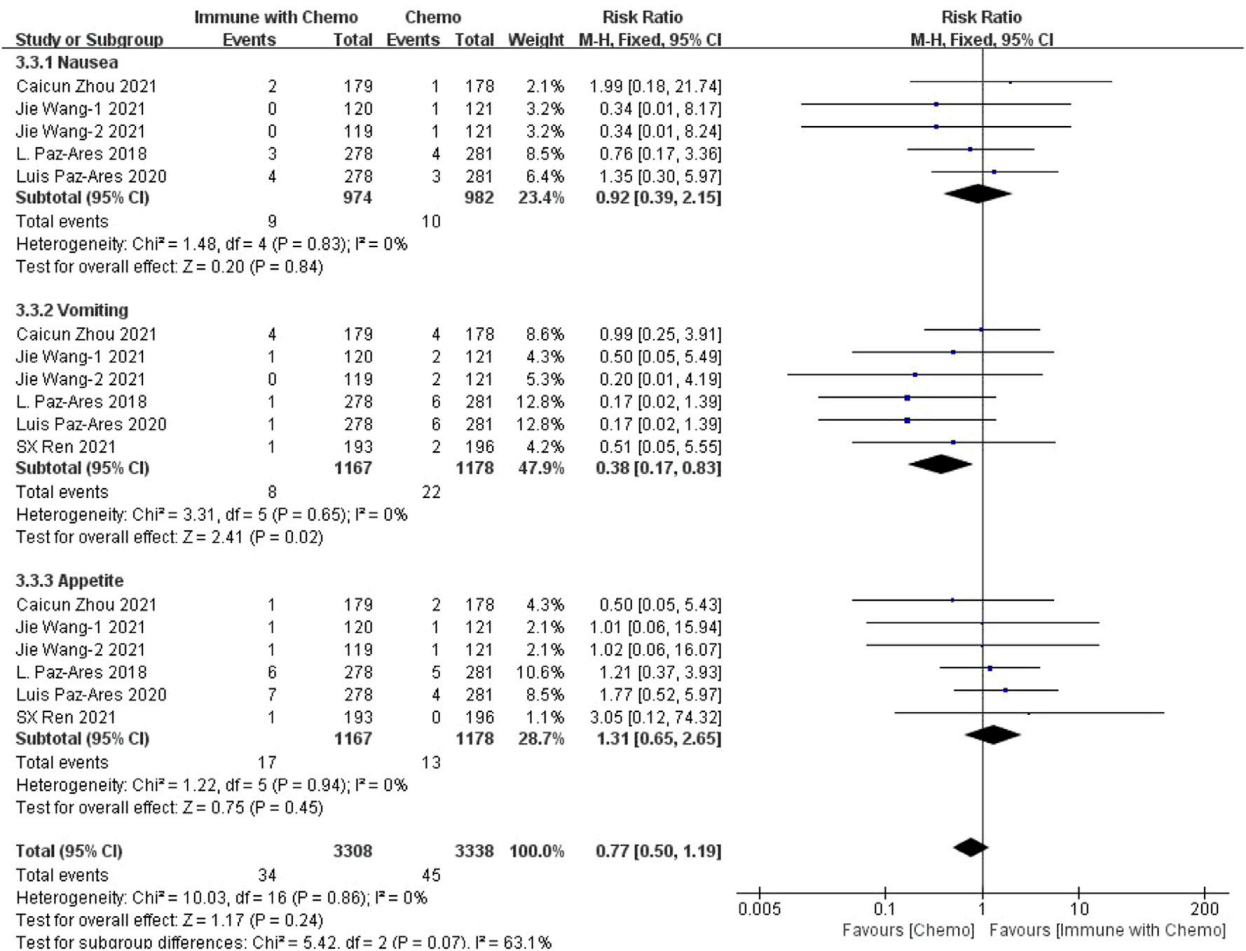
Meta-analysis results of adverse reactions above grade 3 in gastrointestinal toxicity between the Chemo with immune group and chemo group.

Six studies can obtain data on gastrointestinal adverse reactions above grade III (including nausea, vomiting, and appetite) in patients with sq-NSCLC treated by chemotherapy combined with immunotherapy. The heterogeneity is analyzed, with I^2^ = 0%, *p* = 0.86, which is analyzed by the fixed-effect model. The results showed that HR = 0.77 (95% CI = 0.50–1.19, *p* = 0.24), suggesting that the incidence of grade III and above adverse reactions in the digestive system using chemotherapy combined with immunotherapy is relatively high, but there is no significant difference in the overall results, as shown in Figure 7.

#### Meta-Analysis Results of Hepatotoxicity

**FIGURE 8:**
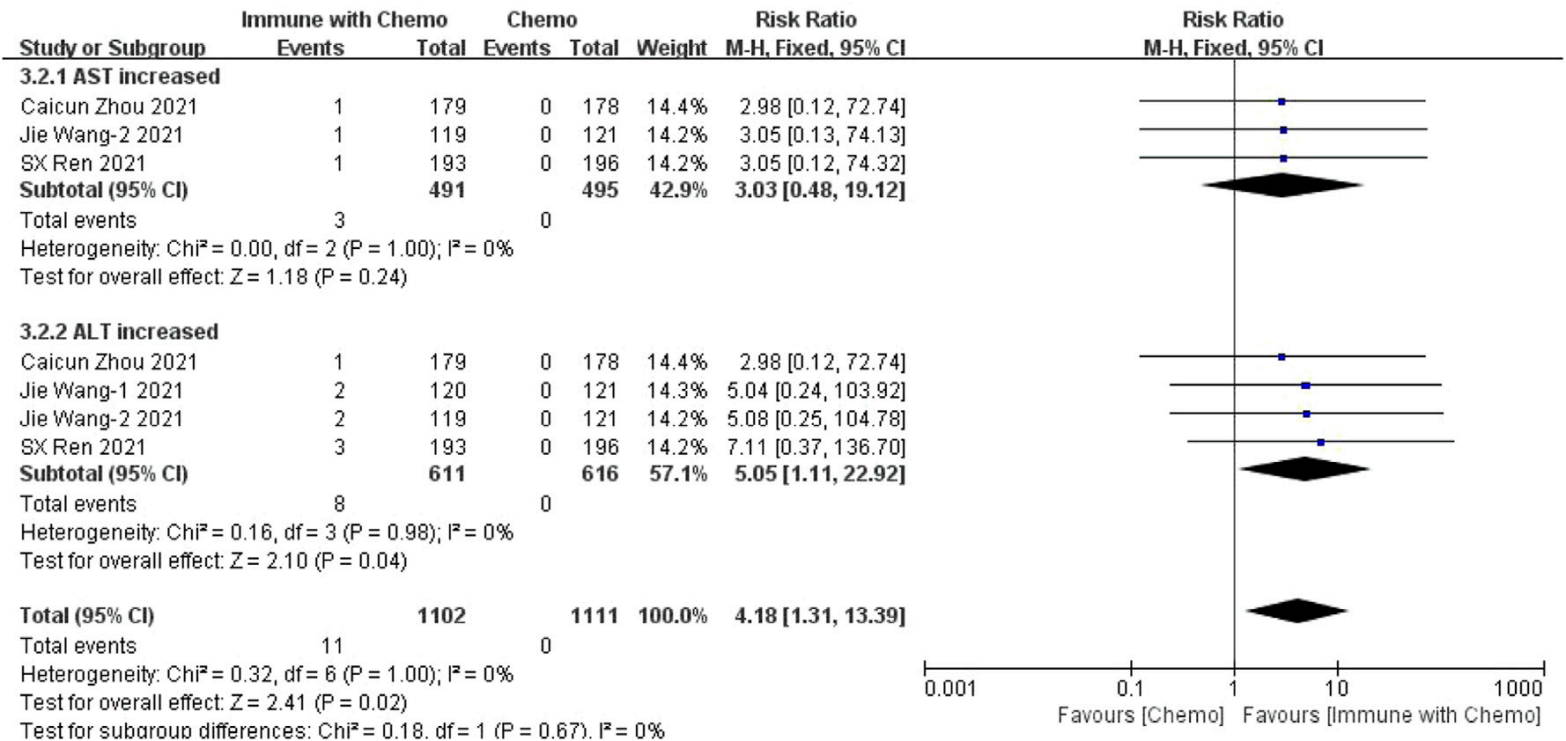
Meta-analysis results of adverse reactions above grade 3 in hepatotoxicity between the Chemo with immune group and chemo group.

Four studies can obtain the data of adverse reactions above grade III of liver function (including AST and ALT) in patients with sq-NSCLC treated by chemotherapy combined with immunotherapy. The heterogeneity is analyzed, with I^2^ = 0%, *p* = 1.00, which is analyzed by the fixed-effect model. The results showed that HR = 4.18 (95% CI = 1.31–13.39, *p* = 0.02), suggesting that the incidence of adverse reactions at any level of liver function using immunotherapy combined with chemotherapy is relatively high, and there is a significant difference in the results, as shown in Figure 8.

### Publication Bias Assessment and Sensitivity Analysis

**FIGURE 9:**
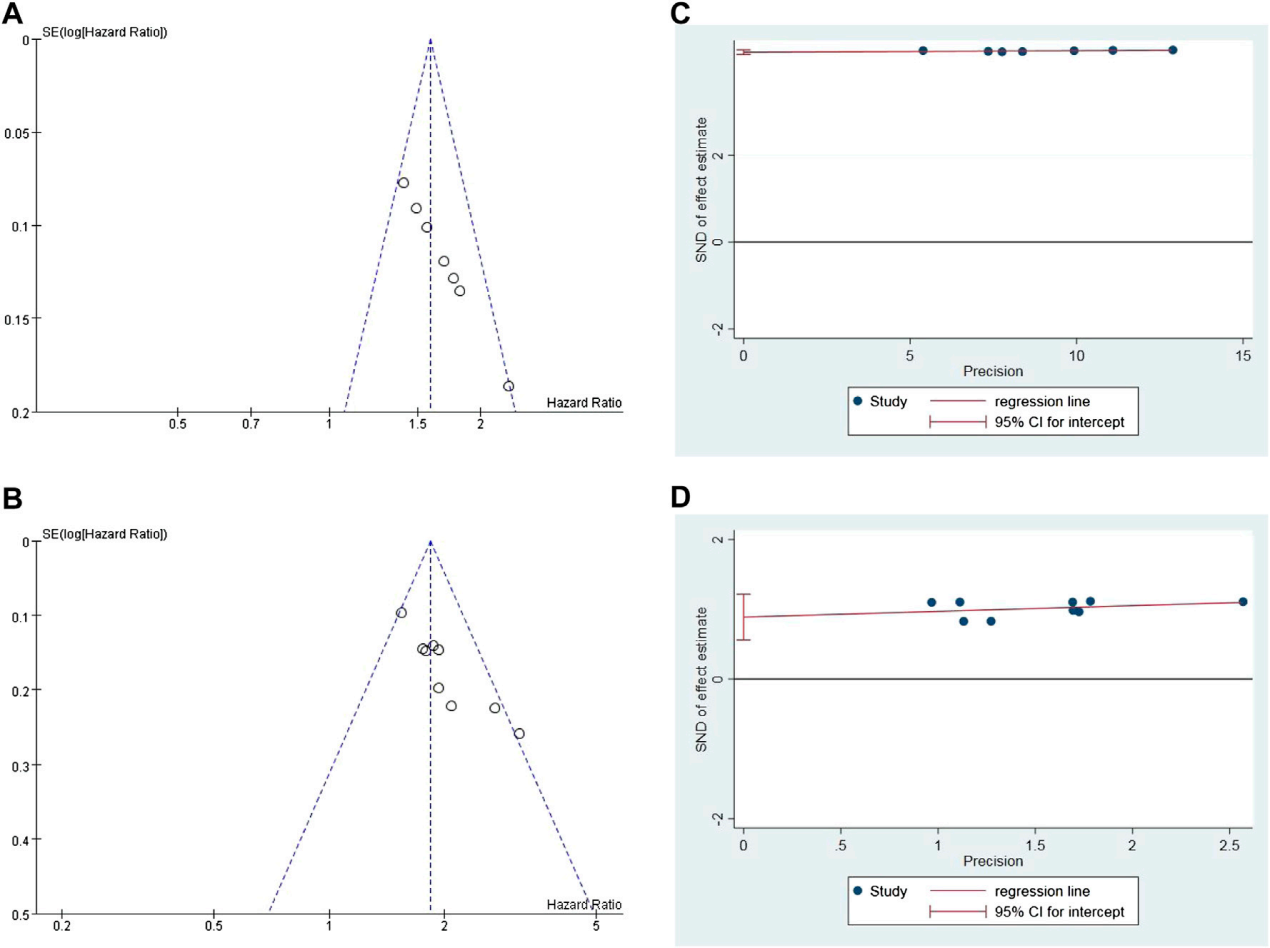
Inverted funnel plot of OS **(A)** and PFS **(B)**. Egger test of OS **(C)** and PFS **(D)**.

Publication bias assessment was performed only in OS and PFS. The funnel plot is symmetrical, indicating no significant publication bias (Figures 9A,B). Sensitivity analysis was conducted on the results, and a meta-analysis was conducted by ignoring each study in turn. No significant changes were found in the results, indicating that the results of this study are stable. Quantitative analysis by Egger’s test showed, Egger test in Stata12 software was used for publication bias test. In a total of nine studies with OS and PFS as outcome indicators, the results of the publication bias test indicated that there was minor publication bias in OS (*p* = 0.0032) and PFS (*p* = 0.0026), as shown in Figures 9C,D. The sources of publication bias in our analysis may be as follows: 1) the number of included studies in meta-analyses is small; 2) the sample size was small; 3) there was heterogeneity in the analysis of individual subgroups; 4) the existence of intra-study publication bias; 5) the observation results of the same group of subjects were divided into multiple articles published by the authors, which may result in multiple publication bias of the subjects. We will continue to include more high-quality literature in the later stages to reduce publication bias.

## Discussion

In recent years, the treatment mode of lung cancer has changed greatly, and the prognosis has improved. The 5-year survival rate of Chinese lung cancer patients diagnosed from 2010 to 2014 has reached 20–30%. Worldwide, the survival rate of lung cancer patients has improved by 5–10% since 2000, while the survival rate of Chinese lung cancer patients has even improved by more than 10% (Allemani et al., 2018). More than 60% of lung adenocarcinomas can find driver genes (The Cancer Genome Atlas Research Network, 2014), including human epidermal growth factor receptor (EGFR), echinoderm microtubule associated protein like 4 (EML4)/anaplastic lymphoma kinase (ALK), and c-ros oncogene 1 receptor tyrosine kinase (ros1) gene fusion. Targeted drugs for the abovementioned targets are constantly updated. The survival rate of patients has significantly improved. However, for lung squamous cell carcinoma, although it accounts for 25–30% of all lung cancers (Houston et al., 2014), the incidence of common driver genes such as EGFR mutation and ALK gene rearrangement is very low, about 2.7 and 1.5–2.5%, respectively (Wang et al., 2014). Therefore, only a few patients with squamous cell carcinoma have the opportunity to receive EGFR tyrosine kinase inhibitor (EGFR-TKI) or ALK inhibitor treatment. The 5-year survival rate of lung squamous cell carcinoma was only 5%. Therefore, sq-NSCLC is a challenging disease with poor prognosis, including tumor location, more complications, and genetic complexity. At present, research shows that some treatment schemes, including new targeted oncogene drugs, chemotherapeutic drugs, and anti-angiogenesis therapy, have limitations in the effectiveness or safety of sq-NSCLC. Therefore, at present, the first-line treatment for the vast majority of sq-NSCLC patients is still platinum-containing combined chemotherapy. Clinical studies have investigated the efficacy and toxicity of cisplatin, carboplatin, and nedaplatin combined with paclitaxel in the treatment of advanced sq-NSCLC (Shukuya et al., 2015). The results showed that the efficiency of cisplatin or carboplatin combined with paclitaxel was significantly higher than that of nedaplatin combined with paclitaxel in the treatment of advanced lung squamous cell carcinoma. At the same time, according to NCCN guidelines, PD-1 inhibitor combined with chemotherapy is recommended as the first-line treatment for patients with advanced squamous NSCLC with PD-L1 > 1%, while PD-1 inhibitor alone is recommended for patients with PD-L1 > 50%. Reviewing that the expression of PD-L1 in the study population is mainly based on patients with less than 1% and more than 50%, only a small number of patients have PD-L1 expression of 1–49%. Therefore, this study does not conflict with NCCN guidelines and has certain clinical guiding significance.

At present, immunotherapy plays an excellent role in prolonging the overall survival and disease-free progression of NSCLC. PD-1/PD-L1 plays an important role in the immune escape of tumor cells. Its pathway exists in the normal immune system. PD-1 is inherently expressed on the surface of activated T lymphocytes, B lymphocytes, and natural killer cells. PD-L1, one of its ligands, is expressed on the surface of tumor cells and tumor-infiltrating immune cells. When PD-L1 is combined with PD-1, it can produce negative stimulation signals and inhibit the activation of T lymphocytes. As a result, T cells cannot engulf tumor cells, and tumor cells escape the monitoring of the immune system, that is, immune escape, which eventually leads to occurrence of tumors. Therefore, PD1/PD-L1 inhibitors activate T lymphocytes to achieve tumor inhibition. PD1/PD-L1 inhibitors commonly used in clinic include sintilimab, tisliezumab, pembrolizumab, camrelizumab, etc., Leena Gandhi et al. (Gandhi et al., 2018) showed that the overall survival rate of pembrolizumab combined with chemotherapy was 69.2% (95% CI, 64.1–73.8), while that of the placebo combined group was 49.4% (95% CI, 42.1–56.2) (HR = 0.49; 95% CI, 0.38–0.64; *p* < 0.01), and the median progression-free survival was 8.8 months (95% CI, 7.6–9.2), 4.9 months (95% CI, 4.7–5.5) in the placebo combination group (HR = 0.52; 95% CI, 0.43–0.64; *p* < 0.001). However, adverse events at level 3 or higher occurred in 67.2% of patients in the pembrolizumab-combined chemotherapy group and 65.8% in the placebo-combined treatment group. Shun Lu et al. (Lu et al., 2021) the open-label phase 3 trial (basic principle 304; nct03663205), patients with nsq-NSCLC were randomly divided into tiselizumab plus platinum (carboplatin or cisplatin) and pemetrexed. The control group was treated with platinum and pemetrexed alone. The results showed that the PFS of the tiselizumab combined chemotherapy group was significantly longer than that of the simple chemotherapy group (median PFS: 9.7 vs. 7.6 months; HR = 0.645 [95% Cl: 0.462–0.902], *p* = 0.0044). In addition, compared with chemotherapy alone, combination therapy has a higher remission rate and longer remission duration.

In this study, we evaluated the efficacy and safety of chemotherapy combined with immunotherapy as the first-line treatment for advanced or metastatic squamous NSCLC, with OS and PFS as the primary outcomes and adverse reactions as the secondary outcome. The disease was limited to the more difficult to cure sq-NSCLC. The results showed that, in terms of effectiveness, chemotherapy combined with immunotherapy improved the HR and *p* value of OS and PFS, indicating that patients receiving immunotherapy combined with chemotherapy had better OS and PFS than patients receiving ordinary chemotherapy. The results were similar to those of other clinical trials. However, in terms of safety, we have refined the adverse reactions into any level of adverse reactions and more than three levels of adverse reactions, mainly including hematotoxicity, hepatotoxicity, and gastrointestinal toxicity. The results showed that 1) in hematotoxicity, the risk ratio of any level of hematotoxicity in the immunotherapy combined with chemotherapy group was higher than that in the chemotherapy group, and the results were significantly different (*p* < 0.00001). In subgroup analysis, there was a significant difference between anemia (*p* = 0.03) and decreased platelet count (*p* = 0.0002), but there was no significant difference between decreased leukocyte count (*p* = 0.25) and decreased neutrophils (*p* = 0.05). There was no significant difference in the incidence of hematotoxicity above grade 3 between the two groups (*p* = 0.30). 2) In hepatotoxicity, the risk ratio of any level of hepatotoxicity in the chemotherapy combined with immunotherapy group was higher than that in the chemotherapy group, and the results were significantly different (*p* = 0.001). Subgroup analysis showed that the increase of AST was more significant in the immune-combined chemotherapy group (*p* = 0.003), and there was no significant difference in ALT between the two groups (*p* = 0.10). For the incidence of hepatotoxicity above grade III, the increase in ALT was more significant in the immune-combined chemotherapy group (*p* = 0.04), and there was no significant difference in AST between the two groups (*p* = 0.24). 3) In gastrointestinal toxicity, the risk ratio of any level of gastrointestinal toxicity in the chemotherapy combined with the immunotherapy group was higher than that in the chemotherapy group, and the results were significantly different (*p* = 0.02). For the incidence of gastrointestinal toxicity above grade 3, there was no significant difference between the two groups (*p* = 0.24), but subgroup analysis showed that the incidence of gastrointestinal toxicity above grade 3 in the combined group was significantly higher and significant (*p* = 0.02). The abovementioned studies suggest that in clinical treatment, chemotherapy combined with immunotherapy as a first-line drug for sq-NSCLC is more effective than chemotherapy alone and can significantly prolong OS and PFS. At the same time, the incidence of adverse reactions is higher than that of chemotherapy. In clinical diagnosis and treatment, we should pay more attention to anemia, changes in platelet count, hepatotoxicity, and some conventional gastrointestinal adverse reactions to treat symptomatic treatment as soon as possible and reduce side effects.

This study also has some limitations: 1) after systematic retrieval and screening, only seven literatures were included for systematic evaluation and meta-analysis, and the sample size is too small; 2) the heterogeneity of individual statistical results may affect the credibility of the research results. This study will continue to update the clinical data and timely supplement the included literature so as to provide a scientific medication basis for the clinical treatment of sq-NSCLC.

## Data Availability

The original contributions presented in the study are included in the article/Supplementary Material; further inquiries can be directed to the corresponding author.
